# PqsBC, a Condensing Enzyme in the Biosynthesis of the *Pseudomonas aeruginosa* Quinolone Signal: CRYSTAL STRUCTURE, INHIBITION, AND REACTION MECHANISM*[Fn FN3]^♦^

**DOI:** 10.1074/jbc.M115.708453

**Published:** 2016-01-25

**Authors:** Steffen Lorenz Drees, Chan Li, Fajar Prasetya, Muhammad Saleem, Ingrid Dreveny, Paul Williams, Ulrich Hennecke, Jonas Emsley, Susanne Fetzner

**Affiliations:** From the ‡ Institute for Molecular Microbiology and Biotechnology and; ‖ Organic Chemistry Institute, University of Münster, D-48149 Münster, Germany and the Centre for Biomolecular Sciences,; Schools of § Pharmacy and; ¶ Life Sciences, University of Nottingham, Nottingham NG7 2RD, United Kingdom

**Keywords:** biosynthesis, crystal structure, Pseudomonas aeruginosa (P. aeruginosa), quorum sensing, secondary metabolism, 2-alkyl-4(1H)-quinolones, FabH, Pseudomonas quinolone signal, condensing enzyme

## Abstract

*Pseudomonas aeruginosa* produces a number of alkylquinolone-type secondary metabolites best known for their antimicrobial effects and involvement in cell-cell communication. In the alkylquinolone biosynthetic pathway, the β-ketoacyl-(acyl carrier protein) synthase III (FabH)-like enzyme PqsBC catalyzes the condensation of octanoyl-coenzyme A and 2-aminobenzoylacetate (2-ABA) to form the signal molecule 2-heptyl-4(1*H*)-quinolone. PqsBC, a potential drug target, is unique for its heterodimeric arrangement and an active site different from that of canonical FabH-like enzymes. Considering the sequence dissimilarity between the subunits, a key question was how the two subunits are organized with respect to the active site. In this study, the PqsBC structure was determined to a 2 Å resolution, revealing that PqsB and PqsC have a pseudo-2-fold symmetry that unexpectedly mimics the FabH homodimer. PqsC has an active site composed of Cys-129 and His-269, and the surrounding active site cleft is hydrophobic in character and approximately twice the volume of related FabH enzymes that may be a requirement to accommodate the aromatic substrate 2-ABA. From physiological and kinetic studies, we identified 2-aminoacetophenone as a pathway-inherent competitive inhibitor of PqsBC, whose fluorescence properties could be used for *in vitro* binding studies. In a time-resolved setup, we demonstrated that the catalytic histidine is not involved in acyl-enzyme formation, but contributes to an acylation-dependent increase in affinity for the second substrate 2-ABA. Introduction of Asn into the PqsC active site led to significant activity toward the desamino substrate analog benzoylacetate, suggesting that the substrate 2-ABA itself supplies the asparagine-equivalent amino function that assists in catalysis.

## Introduction

*Pseudomonas aeruginosa* is a pathogenic Gram-negative bacterium that inhabits soil and aquatic environments. *P. aeruginosa* infections are often nosocomial and usually associated with compromised host defenses (1). The production of many *P. aeruginosa* virulence factors is controlled by quorum sensing (QS),^3^ a mechanism where bacteria communicate by sensing self-generated signaling molecules. QS systems allow bacterial populations to synchronize their behavior and so to act cooperatively (2). Because QS controls the virulence of many pathogenic bacteria, QS receptor function and QS signal generation have been proposed as alternative targets for anti-virulence drug development (3–7).

The QS network of *P. aeruginosa* is highly complex, comprising several signaling circuits that are interconnected (8). The Las and Rhl circuits use specific *N*-acylhomoserine lactones as signal molecules, whereas the Pqs circuit employs the alkylquinolone (AQ) signals 2-heptyl-4(1*H*)-quinolone (HHQ) and 2-heptyl-3-hydroxy-4(1*H*)-quinolone (*Pseudomonas* quinolone signal (PQS)). AQ signaling influences biofilm development and the production of a number of virulence factors, such as pyocyanin, siderophores, rhamnolipid biosurfactant, the cytotoxic lectin LecA, and elastase LasB (9, 10). Moreover, PQS induces membrane vesicle formation (11), acts as ferric iron chelator (12, 13) and pro-oxidant (14), and exerts host immune modulatory and pro-apoptotic activities (15–17).

**FIGURE 1.**
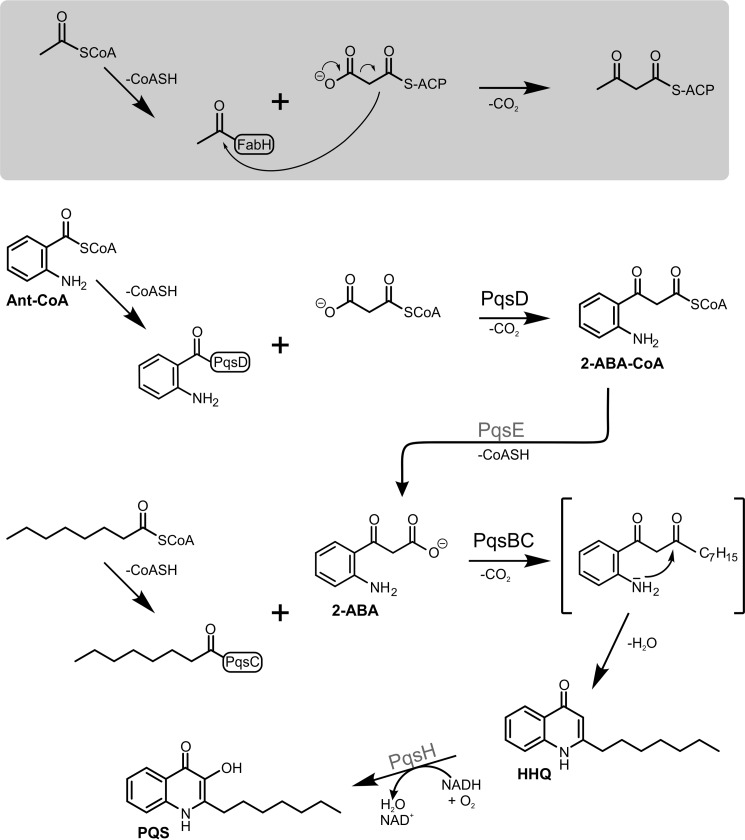
**Reactions of FabH (*shaded box*) and the FabH-like proteins PqsD and PqsBC involved in 2-alkyl-4(1*H*)-quinolone biosynthesis.** The PqsD- and PqsBC-catalyzed reactions proceed in a similar manner to that of the model enzyme FabH. In the initial step, the acyl moiety of an activated carboxylic acid is transferred to a strictly conserved active-site cysteine residue. Subsequently, a β-ketoalkanoic acid (malonyl-ACP in case of FabH, malonyl-CoA or malonyl-ACP in PqsD, and 2-ABA in PqsBC) is decarboxylated, and the resulting reactive enolate intermediate (not shown) attacks the thioester bond of the acyl-enzyme to form the reaction product. Intermediates and products of the alkylquinolone biosynthetic pathway: *Ant-CoA*, anthraniloyl-coenzyme A; *2-ABA-CoA*, 2-aminobenzoylacetyl-CoA; *2-ABA*, 2-aminobenzoylacetate; *HHQ*, 2-heptyl-4(1*H*)-quinolone; *PQS*, *Pseudomonas* quinolone signal.

Biosynthesis of HHQ and PQS from anthranilic acid and fatty acid precursors is initiated by the coenzyme A (CoA) ligase PqsA, which catalyzes the activation of anthranilic acid to anthraniloyl-CoA (18). A subsequent condensation reaction with malonyl-CoA, catalyzed by PqsD, yields the highly unstable intermediate 2-aminobenzoylacetyl-CoA (2-ABA-CoA); however, PqsD also uses malonyl-acyl carrier protein (ACP) as substrate (19). Although 2-ABA-CoA is highly susceptible to spontaneous cyclization to form 2,4-dihydroxyquinoline (DHQ) (19–21), *in vivo* this is counterbalanced by the activity of PqsE, which acts as a 2-ABA-CoA thioesterase to release 2-aminobenzoylacetate (2-ABA) (21). 2-ABA is another branching point in the pathway and can undergo decarboxylation to 2-aminoacetophenone (2-AA) (20), a secondary metabolite reported to promote chronic infection phenotypes of *P. aeruginosa* (22, 23) and to modulate the host innate immune response (24, 25). Alternatively, it can be channeled into HHQ biosynthesis by the activity of PqsBC, which catalyzes the condensation of 2-ABA and octanoyl-CoA to form HHQ (Fig. 1) (20, 21). Hydroxylation of HHQ to PQS is catalyzed by the flavin monooxygenase PqsH (26).

PqsD is a member of the FabH family of condensing enzymes (27). PqsC and PqsB, which form a tight complex (20, 21), also have been annotated to belong to this family. FabH (β-ketoacyl-ACP synthase III), a key enzyme in fatty acid biosynthesis, catalyzes the decarboxylating condensation of acetyl-CoA and malonyl-ACP to form acetoacetyl-ACP (Fig. 1). The active site of *Escherichia coli* FabH is composed of residues Cys-112, His-244, and Asn-274, which are required to catalyze the complete condensation reaction. Cys-112 is loaded with the acetyl residue in the initial step of the reaction, whereas His-244 and Asn-274 are involved in the subsequent decarboxylation reaction of malonyl-ACP (28). To identify the determinants of catalysis, to gain insight into the PqsBC reaction mechanism, and to probe possible effects of AQ metabolites on PqsBC activity, we performed the first structural and mechanistic investigation of this unique condensing enzyme. FabH-like enzymes are usually homodimers in solution; however, the PqsBC crystal structure we report here reveals that PqsB and PqsC form a novel heterodimer. PqsC lacks the conserved asparagine, and PqsB lacks all three conserved residues of the FabH catalytic triad, raising the question of how PqsBC mediates the condensation reaction.

## Experimental Procedures

### 

#### 

##### Expression and Purification of PqsBC

The *pqsB* and *pqsC* genes of *P. aeruginosa* PAO1 were cloned into plasmid pET28b using restriction-free cloning (29). In the process, *pqsC* was fused with the sequences encoding a short linker (Ser-Ala-Gly), a recognition site for tobacco etch virus (TEV) protease, and an N-terminal octahistidine tag. The vector-internal sequences for affinity tags were not used. The coding sequence of the resulting plasmid pET28b::*pqsBC*-NHis_8_ was verified using commercial sequencing services. Site-specific mutations within the *pqsC* gene were introduced using restriction-free cloning (29). For protein overproduction, *E. coli* Rosetta2(DE3)[pLysS] carrying the respective expression plasmid was grown in TB medium (Carl Roth, Karlsruhe, Germany) supplemented with 150 μg ml^−1^ kanamycin (Applichem, Darmstadt, Germany). Cells were cultivated in baffled flasks at 37 °C until reaching a cell density (*A*_600_) of 0.8 and then cooled to 20 °C before gene expression was induced with 0.2 mm isopropyl β-d-thiogalactopyranoside (Thermo Scientific, Waltham, MA). After 8–12 h, cells were harvested by centrifugation, flash-frozen in liquid N_2_, and stored at −80 °C until use.

For the preparation of PqsBC, cells were resuspended in 20 mm Tris-HCl buffer, pH 8.0, containing 150 mm NaCl, 10 mm imidazole, 5 mm EDTA, 0.5 mm β-mercaptoethanol, and 0.1% Nonidet P-40 (all Carl Roth, Karlsruhe, Germany). Cells were disrupted by sonication (Hielscher UP200S Hielscher, Germany), and subsequently, cell debris was removed by centrifugation for 1 h at 50,000 × *g* at 4 °C. Purification of PqsBC was performed using immobilized metal affinity chromatography, anion exchange, and gel filtration chromatography. All chromatographic procedures were carried out using either Äkta PrimePlus (GE Healthcare) or BioLogic (Bio-Rad) chromatography systems. To this end, cell extract supernatants were filtered (0.45 μm polyvinylidene fluoride membrane, Carl Roth, Karlsruhe, Germany) and applied onto a 5-ml nickel-nitrilotriacetic acid-Sepharose column (Qiagen, Hilden, Germany) equilibrated with a wash buffer containing 20 mm Tris, pH 8.0, 150 mm NaCl, and 10 mm imidazole. The column was washed with 10 column volumes (CV) of wash buffer before a 10-CV gradient against an elution buffer containing 20 mm Tris, 150 mm NaCl, and 150 mm imidazole was applied. Protein-containing fractions were pooled and dialyzed at room temperature against a buffer containing 10 mm Tris and 0.5 mm DTT (Carl Roth, Karlsruhe, Germany) using a 14-kDa molecular mass cutoff dialysis tube (Servapor, Serva Electrophoresis, Heidelberg, Germany). After 2 h, TEV protease (see below; 1 μg of TEV protease per 20 μg of total protein), arginine, and glutamate (final concentration 50 mm, Sigma) were added directly to the dialysis tube, and the tube was transferred into fresh buffer. Dialysis was continued overnight at 4 °C with gentle stirring. Precipitated material was removed by centrifugation at 50,000 × *g* (20 min at 4 °C), and the supernatant was passed through a 1-ml HisTrap column (GE Healthcare) equilibrated with 20 mm Tris-HCl, pH 8, to separate PqsBC from the uncleaved fusion protein, TEV protease, and His tag peptides. The flow-through was collected and subsequently applied to a 6-ml Resource Q column (GE Healthcare) equilibrated with a wash buffer containing 15 mm MOPS, pH 8.1 (Carl Roth, Karlsruhe, Germany), and 1 mm DTT. The column was washed with 20-CV wash buffer before a linear 20-CV gradient against 15 mm MOPS, pH 8.0, 400 mm NaCl, 1 mm DTT was started. PqsBC-containing fractions were pooled and passed through a second 1-ml HisTrap column equilibrated with a buffer containing 15 mm MOPS, pH 8.0, 150 mm NaCl to remove residual impurities. The flow-through was collected and concentrated to a volume of ∼1.5 ml using a 10-kDa molecular mass cutoff centrifugal concentrator (Sartorius, Goettingen, Germany). The protein solution was applied onto a HiLoad26/60 Superdex 200 gel filtration column (GE Healthcare) equilibrated with a buffer containing 40 mm MOPS, pH 8.1, and 150 mm NaCl. For molecular weight estimation, a calibration run using a Bio-Rad gel filtration standard was used. Elution fractions containing the PqsBC protein were pooled, and 2 mm DTT was added. The solution was concentrated to >20 mg ml^−1^ and applied onto two serially connected 5-ml HiTrap buffer exchange columns (GE Healthcare) equilibrated with the storage buffer containing 40 mm MOPS, pH 8.1, and 50 mm NaCl. Eluted protein fractions were pooled, aliquoted, flash-frozen, and stored at −80 °C until required for further use. Protein concentration was determined by UV spectroscopy according to Gill and von Hippel (30) using a calculated extinction coefficient of 72,880 m^−1^ cm^−1^. Purification of PqsBC protein variants followed the same protocol.

##### Expression and Purification of Tobacco Etch Virus Protease

The L56V/S135G/S219V variant of TEV protease was overproduced and prepared as described by Cabrita *et al.* (31).

##### Synthesis of 2-Aminobenzoylacetate (2-ABA)

In a 250-ml flask, 2-nitrobenzoylacetate (32–34) (84 mg, 0.40 mmol) was dissolved in water (10 ml) and aqueous ammonia (0.1 ml, ∼25 weight % in water). Palladium on charcoal (75 mg, 5% Pd/C, Sigma) was added, and the flask was placed under a hydrogen atmosphere (atmospheric pressure, by three cycles of brief evacuation and backfilling with hydrogen from a balloon). The suspension was rapidly stirred for 40 min under hydrogen and then filtered through a pad of Celite. The solvent was removed under high vacuum to give the product (56 mg, 0.31 mmol, 78%) as a yellow-green gum. ^1^H NMR spectra were recorded on an Agilent DD2 600 spectrometer (600 MHz). ^13^C NMR spectra were recorded on an Agilent DD2 600 spectrometer (150 MHz). Chemical shifts δ are given in ppm and are referenced according to IUPAC recommendations on a unified chemical shift scale for all nuclides based on the proton resonance of tetramethylsilane as primary reference. MS-ESI(+) spectra were recorded on a Thermo Scientific^TM^ Orbitrap LTQ XL using nanospray methods. Data were as follows: ^1^H NMR (600 MHz, D_2_O, 299 K): δ = 7.67–7.62 (m, 1H), 7.26–7.21 (m, 1H), 6.72–6.68 (m, 1H), 6.65–6.60 (m, 1H), 3.71/3.69 (s, the intensity of these signals, which represents the -CH_2_- and -CHD- group depends strongly on the time between dissolution and measurement of the spectrum due to the slow exchange of hydrogen against deuterium). ^13^C NMR (150 MHz, D_2_O, 299 K): δ = 200.0, 175.9, 150.5, 135.3, 132.1, 118.0, 117.9, 116.7, and 49.9–49.4 (m, overlay of signals of CH_2_-, -CHD-, and -CD_2_- groups). MS-ESI(+): *m/z* = 403 (30) [2M − H + 2Na]^+^, 381 (60) [2M + Na]^+^, 218 (25) [M + K]^+^, 202 (100) [M + Na]^+^, 180 (55) [M + H]^+^, 162 (30) [M − H_2_O + H]^+^. MS-ESI(+)-EM: *m/z* = 202.04746 calculated for C_9_H_9_NO_3_Na [M + Na]^+^ and found 202.04705.

##### Spectrofluorimetric Determination of Substrate and Inhibitor Binding to PqsBC

Binding equilibria as well as octanoylation of PqsBC were measured by spectrofluorimetric titration using Jasco FP-6500 or Jasco FP-8300 instruments (Jasco, Tokyo, Japan) and 100-μl quartz cuvettes. All measurements were performed in ratio mode with a 5-nm excitation/emission bandwidth, 1-s response, and manually adjusted photomultiplier sensitivity. Titrants were added with an eVol digital analytical syringe (SGE/Trajan Scientific, Melbourne, Australia). For determination of dissociation constants for the PqsBC-2-ABA and PqsBC-2-AA complexes, the fluorescence properties of the substrates were utilized (λ_Ex_ = 359 nm and λ_Em_ = 470 nm). Assays were carried out in 50 mm HEPES, pH 8.2, and 50 mm NaCl at 22 °C, titrating 2-ABA or 2-AA (0–3 mm) into PqsBC (1–10 μm) or vice versa (0–300 μm PqsBC, 1–10 μm 2-ABA or 2-AA). After every titration step, the mixture was equilibrated for 1 min before the measurement was taken. Recorded spectra were corrected for dilution and inner filter effects as described elsewhere (35). For detection of covalent octanoate binding, 2-AA was used as a fluorescence probe. The same conditions as above were used, but 2-AA (0–50 μm) and PqsBC (0–50 μm) were premixed, and octanoyl-CoA (Sigma) was added while fluorescence was monitored over time (0.5 s resolution, 10 s mixing time). All experiments were carried out with at least three technical replicates. To exclude variance between protein preparations, four different batches of PqsBC were assayed. Any influence of the affinity tag on substrate or 2-AA binding could be excluded, because titrations with tagged and protease-processed proteins gave similar binding constants.

Equilibrium kinetic data from spectrofluorimetric titrations were fitted using Matlab R2014b with optimization toolbox (The Mathworks). For titrations of 2-ABA or 2-AA with PqsBC, the concentration of the enzyme in complex with substrate or substrate analog was calculated from the law of mass action as shown in Equation 1,

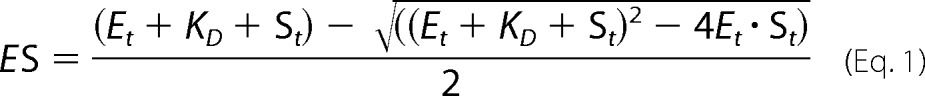
 where *E*S is the concentration of the enzyme-substrate complex; *E_t_* is the total enzyme concentration; *K_D_* is the dissociation constant, and S*_t_* is the total substrate concentration. Fluorescence data were converted to binding ratios using the expression given in Equation 2,

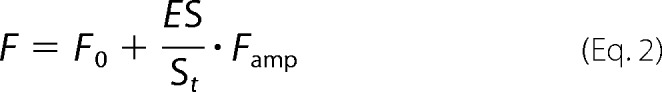
 when S*_t_* was kept constant, or Equation 3 when *E_t_* was kept constant in the assay.

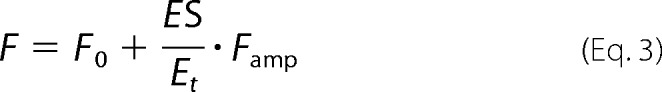


*F* is the measured fluorescence; *F*_0_ is the starting fluorescence value, and *F*_amp_ is total amplitude of fluorescence change.

##### Transient Kinetics of PqsBC Octanoylation

Stopped-flow fluorimetry was used to measure the transient kinetics of PqsBC octanoylation. Experiments were conducted using an SFM-3 stopped-flow instrument (Biologic, Claix, France) coupled to a Jasco FP-6500 fluorimeter. 2-AA or 2-ABA (120 μm) was mixed with PqsBC protein (30 μm), octanoyl-CoA (120 μm), and 2-AA or 2-ABA (120 μm) from separate reservoirs. Fluorescence changes of 2-AA or 2-ABA upon protein binding were used to probe octanoylation. Samples were excited at 360 nm, and emission was measured at 450 nm (bandwidth 10 nm). The minimal detector response time was 10 ms, and the stopped-flow unit was operated at maximum speed (dead time 1.8 ms). 10–20 experiments were averaged for data collection. Data were fitted using a single exponential function, and rate constants were calculated assuming a first order reaction.

##### Enzyme Activity Assays with 2-ABA and Octanoyl-CoA

Catalytic activity of PqsBC was measured with 2-ABA and octanoyl-CoA. Assays were performed at 25 °C in buffer containing 50 mm HEPES, pH 8.2, 50 mm NaCl, and 100 μm DTT (all from Carl Roth, Karlsruhe, Germany). Discontinuous assays were based on the quantification of HHQ by HPLC formed in the reaction as described previously (21). Continuous spectroscopic assays were based on measuring either 2-ABA consumption or HHQ formation. The extinction coefficient of 2-ABA, quantified by the amount of DHQ formed upon acidification, was 5,025 m^−1^ cm^−1^ at 360 nm (in assay buffer, pH 8.2). For setting up a spectroscopic assay detecting HHQ, defined amounts of 2-ABA were fully converted by PqsBC with an excess of octanoyl-CoA, and the resulting HHQ was quantified by HPLC. UV spectra were recorded before and after the reaction (Jasco V-650 spectrophotometer), which, in combination with the known UV-visible spectral properties of HHQ and 2-ABA, allowed the estimation of a differential extinction coefficient for the HHQ formation of ϵ_313 nm_ = 6,520 m^−1^ cm^−1^. However, the value at concentrations of more than 10 μm HHQ is meaningless due to scattering effects caused by precipitating HHQ. The apparent steady-state kinetic constants of PqsBC were estimated by fitting the initial velocities measured at different substrate concentrations with the Michaelis-Menten equation. The assays contained 0.02 μm PqsBC, and 2-ABA was varied from 10 to 500 μm at 100 μm octanoyl-CoA, whereas octanoyl-CoA was varied at concentrations between 1 and 200 μm at 500 μm 2-ABA. For testing the inhibitory potential of 2-AA (Sigma), DHQ (Ferak, Berlin, Germany), and PQS and HHQ (both from Sigma) on 2-ABA conversion by PqsBC, the assays contained 50 μm octanoyl-CoA and varying concentrations of 2-ABA and the potential inhibitor. EC_50_ values were calculated by non-linear fitting using the appropriate logistic function. Inhibition constants were either determined graphically from the Dixon plot (smallest Euclidean distance between regression lines) or calculated by least squares fit over all measured initial velocities using Equation 4,

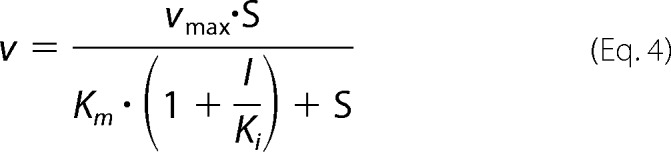
 where *v* is the measured initial reaction velocity; *v*_max_ is the maximum velocity; S is the initial substrate concentration; *K_m_* is the apparent Michaelis constant without inhibitor added; *I* is the inhibitor concentration, and *K_i_* is the competitive inhibition constant. All data processing, fitting, and calculations were performed with MATLAB R2014A with curve fitting and optimization toolboxes (The Mathworks, Natick, MA).

##### Activities of PqsBC with Substrate Analogs

Because the reactivity of PqsBC with octanoyl-CoA and substrate analogs benzoylacetate (BA) or malonyl-CoA cannot be analyzed by spectral changes upon product formation, a colorimetric assay based on the reaction of Ellman's reagent with free thiols originating from transacylation of octanoyl-CoA was employed (36). For assaying the activity of PqsBC with octanoyl-CoA and BA, 2.5 mm BA and 250 μm octanoyl-CoA were mixed in a buffer containing 50 mm HEPES, pH 8.2, and 50 mm NaCl before PqsBC (concentration between 0.001 and 1 μm) was added. The sample volume was 50 μl. Samples were incubated between 1 and 30 min before reactions were stopped by adding 48 μl of 200 mm Tris, pH 7.4, 1% SDS. The chromogenic reaction was started by adding 2 μl of 50 mm 5,5′-dithiobis-(2-nitrobenzoic acid) (DTNB). Sample absorbances were immediately measured at 412 nm (ϵ_DTNB_ = 13,700 cm^−1^ m^−1^ (36)). All sample data were corrected against equivalently treated samples in which the enzyme was inactivated with 0.5% SDS before adding it to the assay mixture. Assays with octanoyl-CoA and malonyl-CoA were conducted analogously, using 100 μm octanoyl-CoA and 250 μm malonyl-CoA. Samples were incubated for 1 h before reactions were stopped, and free thiol content was measured.

##### Analysis of the in Vivo Inhibition of HHQ Biosynthesis by 2-AA

*Pseudomonas putida* KT2440 [pBBR-*pqsABCD-his*], when grown in mineral salts medium supplemented with anthranilic acid, produces substantial amounts of HHQ (37). To test the effect of 2-AA on the *in vivo* HHQ synthesis, the recombinant strain was grown as described previously (37) in the presence of different concentrations of 2-AA. Culture samples were withdrawn in 30–120-min intervals and analyzed by HPLC for HHQ contents. Possible effects of 2-AA on the production of the biosynthetic enzymes were monitored by Western blotting and immunodetection of the His_6_-tagged PqsD protein using a pentahistidine-specific monoclonal antibody (Qiagen, Hilden, Germany).

##### Crystallization and Structure Determination of PqsBC^C129A^

Protein samples of wild-type PqsBC and the active site protein variant PqsBC^C129A^ in 40 mm MOPS buffer, pH 8.1, 100 mm NaCl, 0.3 mm DTT at protein concentrations of 31.5 and 36.5 mg ml^−1^, respectively, were diluted with 100 mm NaCl to give 3 mg/ml. These samples were subjected to screens, including Pi-PEG, Procomplex, classics lite, Mbclass, Index, PACT, Morpheus, JCSG+, and MIDAS, using the sitting drop vapor diffusion method (Mosquito robot, TTP Labtech, Melbourne, UK). Crystals of PqsBC^C129A^ resulted from the conditions of 0.2 m NaCl, 0.1 m Tris, pH 8.5, 25% PEG 3350 within 24 h, whereas octanoylated or apo-forms of wild-type PqsBC did not form crystals. Optimization was performed using 24-well sitting drop plates varying PEG in 1% increments, 23–27%, as well as Tris, pH 8.3–8.7. The final optimized crystallization condition was 25% PEG 3350, 0.1 m Tris, pH 8.3, and 0.2 m NaCl with 3 mg ml^−1^ protein concentration and a total volume of 4 μl. PqsBC^C129A^ crystals were cryoprotected in a solution of 27% PEG 3350, 0.1 m Tris, pH 8.3, 0.2 m NaCl, 25% glycerol, and flash-cooled in liquid nitrogen prior to data collection at the Diamond Light Source synchrotron facility beamline I02 to 2.0 Å resolution with space group P2_1_2_1_2_1_ and a unit cell of *a* = 78.5 Å, *b* = 115.0 Å, and *c* = 289.7 Å, α = 90°, β = 90°, and γ = 90° (Table 1). This unit cell was predicted to have four PqsB and four PqsC polypeptides in the asymmetric unit with ∼50% solvent content. Molecular replacement was performed with PHASER using a variety of templates generated using MrBUMP (38) from PDB codes 3h76, 3h77, and 3h78 of the PqsD structure (39). Using different resolution cutoffs, this resulted in an incomplete solution from which a partial model was built using BUCCANEER (40), and electron density maps were subsequently improved using 4-fold non-crystallographic symmetry and solvent flattening with the CCP4 programs DM (41) and PARROT (42). The resulting density-modified 2 Å electron density map was of high quality allowing 95% of the model to be built with BUCCANEER. The model was completed manually using COOT and refined with REFMAC5 (43) giving 2,474 residues in total, and crystallographic statistics are listed in Table 1 (PDB code 5DWZ). Values for surface area buried were calculated using PISA (44).

**TABLE 1:** **Data collection and refinement statistics**

Data collection	PqsBC^C129A^
Space group	P2_1_2_1_2_1_
*a*, *b*, *c* (Å)	78.5, 115.0, 289.7
α, β, γ (°)	90, 90, 90
Wavelength	0.97949
Resolution (Å)	2.0
*R*_merge_*^a,b^*	0.069 (0.696)
*I*/σ*I*	12.5 (1.9)
Completeness (%)	99.2 (96.7)
Redundancy	4.5 (4.6)
CC 1/2*^c^*	0.998 (0.661)
Unique reflections	166,566
*R*_work_*^d^*	0.190 (0.291)
*R*_free_	0.237 (0.321)
Overall *B* factor (A^2^)	38.1
Bond lengths (Å)	0.016
Bond angles (°)	1.81
Most favored (%)*^e^*	97.7
Allowed (%)	2.3
Outliers (%)	0

*^a^* Values in parentheses correspond to the highest resolution shell.

*^b^ R*_merge_ = Σ*h* Σ*i*|*Ii*(*h*) − 〈*I*(*h*)〉/|Σ*h* Σ*i Ii*(*h*), where *I* is the observed intensity, and 〈*I*(*h*)〉 is the average intensity of multiple observations from symmetry-related reflections calculated with XDS.

*^c^* Correlation coefficient value was calculated using XDS to determine the resolution cutoff.

*^d^* All values were calculated using REFMAC. *R*_work_ = Σ*h* ‖*Fo*|*h* − |*Fc*|*h*|/Σ*h*|*Fo*|*h*, where *Fo* and *Fc* are the observed and calculated structure factors, respectively. *R*_free_ computed as in *R*_work_, but only for (5%) randomly selected reflections, which were omitted in refinement.

*^e^* Ramachandran plot is shown.

## Results

### 

#### 

##### Synthesis and Stability of 2-ABA and Benzoylacetate

Quantitative investigation of the PqsBC reaction requires highly purified substrates applicable at defined concentrations; however, 2-ABA was previously described as relatively unstable in solution, decomposing to 2-AA and also to DHQ (20, 34). After testing a series of conditions, we discovered that hydrogenation in an alkaline aqueous solution is the key to obtain 2-ABA in high yield and purity. Using dilute ammonia solution as medium for hydrogenation of the 2-nitrobenzoylacetate precursor, we obtained 2-ABA in 78% yield with ∼95% purity (as indicated by HPLC and NMR data). In aqueous solution at a pH above 8, it is stable for several days at room temperature (in the dark) and can be stored at −80 °C for months. When solutions of 2-ABA were acidified, the compound decomposed to DHQ. However, when exposed to organic solvents such as methanol, acetonitrile, or dimethyl sulfoxide, rapid decarboxylation to 2-AA as the sole decomposition product was observed. BA, synthesized as described previously (21), was used as an analog of 2-ABA in the PqsBC reaction. It turned out to be unstable in aqueous solution at neutral pH and decarboxylated within minutes to hours, yielding acetophenone.

##### PqsBC^C129A^ Crystal Structure Reveals a Heterodimer

**FIGURE 2.**
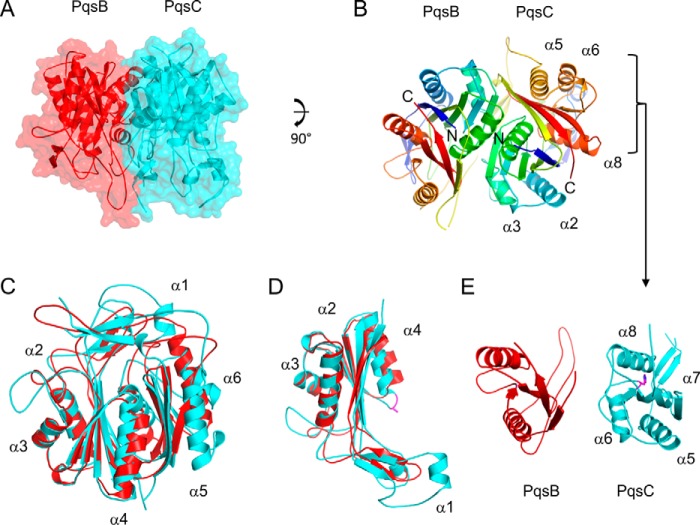
**Schematic diagrams of the PqsBC^C129A^ structure.** *A,* PqsBC^C129A^ heterodimer is shown with PqsC (*cyan*) and PqsB (*red*) covered with a transparent molecular surface illustrating the interface. *B,* PqsB and PqsC^C129A^ structures are colored in a rainbow from the N (*blue*) to the C terminus (*red*) with secondary structure elements labeled for PqsC^C129A^ in all panels. *C,* superposition of PqsB and PqsC^C129A^. *D,* N-terminal sub-domains of PqsB(1–154) and PqsC(1–184) superposed with the catalytic dyad residue Ala-129 shown as sticks (*purple*). *E,* C-terminal sub-domains of PqsB(155–279) and PqsC(188–348) arranged side by side viewed with the same orientation showing PqsC^C129A^ helix α6 is missing in PqsB. PqsC residue His-269 is shown as sticks (*purple*).

PqsBC^C129A^ crystallized in space group P2_1_2_1_2_1_ and the structure was determined to 2 Å resolution with the final model refined to crystallographic *R*-factors of *R*_work_ = 0.190 and *R*_free_ = 0.237 (Table 1). The crystallographic asymmetric unit contains four copies of PqsB and four copies of PqsC. An analysis of the interfaces reveals a large ∼2,238 Å^2^ surface area buried by interactions formed between the PqsB and PqsC subunits as shown in Fig. 2*A*. This is consistent with gel filtration data indicating that PqsB and PqsC form a tight complex with an apparent molecular mass of 51 kDa. The structure is thus referred to as the PqsBC^C129A^ heterodimer. This large interface between PqsB and PqsC is observed to be almost identical in the four copies observed in the asymmetric unit (values of buried surface area are 2,277, 2,240, 2,223, and 2,212 Å^2^ averaged to 2,238 Å^2^), and the heterodimers superimpose onto each other well with an average root mean square deviation of 0.243 Å (8,182 atoms).

The arrangement of secondary structural elements colored from the N to the C termini shows PqsB and PqsC assemble into a dimer with the N termini (*blue*) co-localizing close to the pseudo 2-fold axis and the C termini (*red*) being placed adjacent directed away from the axis (Fig. 2*B*). The fold of PqsC and PqsB is closely related to the FabH enzymes and PqsD with two subdomains each composed of a four-stranded β-sheet surrounded by α-helices. The structural superposition of PqsB and PqsC shown in Fig. 2*C* is largely driven by similarities in the N-terminal subdomain (Fig. 2*D*). Substantial differences occur in the C-terminal subdomains with PqsB having a shorter sequence (124 amino acids) with only two α-helices and PqsC (160 amino acids) having four α-helices (Fig. 2*E*). The large difference in the amino acid sequence of the PqsB C-terminal region compared with PqsC means that it is difficult to align the primary sequences, and there is no identifiable catalytic triad or dyad in the PqsB structure indicating it likely contributes a supporting structural role to the enzyme functionality.

##### PqsC^C129A^ Structure Defines a Catalytic Dyad

**FIGURE 3.**
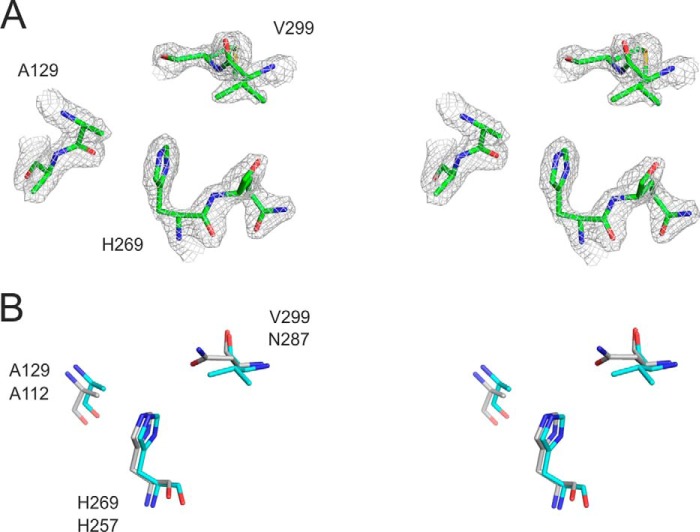
**Stereo figures to illustrate the PqsBC^C129A^ catalytic dyad.** *A,* 2.0 Å electron density map is shown (*gray*) contoured at 2.0 σ level calculated using the final refined weighted 2*Fo* − *Fc* coefficients (REFMAC) and rendered with PyMOL showing PqsC residues Ala-129, Ala-130, His-269, Gln-270, Val-299, and Met-300 as *sticks* in *green. B,* PqsBC^C129A^ Ala-129, His-269 catalytic dyad is shown in *cyan* superposed with the PqsD^C112A^ triad residues (*gray*).

**FIGURE 4.**
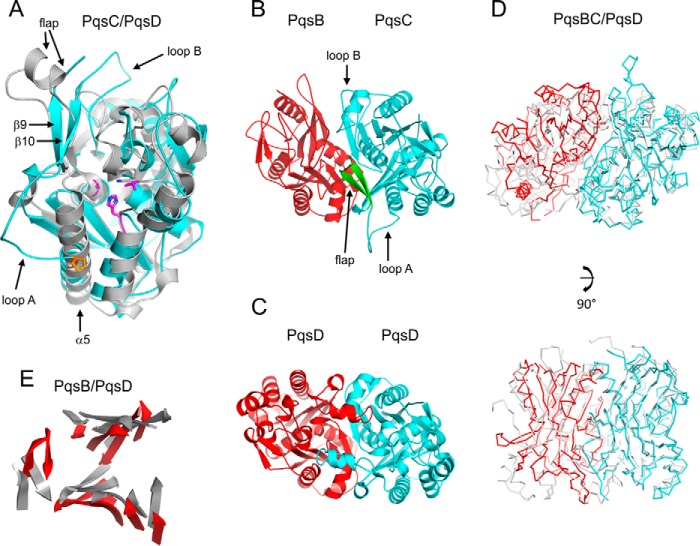
**Comparison of PqsD and PqsBC^C129A^.** *A,* PqsC^C129A^ structure is shown as a schematic (*cyan*) superposed onto PqsD (*gray*). Residues from PqsC shown as *sticks* in *purple* are Ala-129, His-269, Val-299, and Pro-242 (*orange*). PqsD residues Ala-112, His-257, and Asn-287 are shown as sticks (*gray*). *B,* schematic diagram of PqsBC (*red, cyan*) with the flap (PqsC residues 207–227) in *green* shown in the same orientation as the PqsD homodimer below (*C*). *D,* superposition of the PqsBC structure (*red, cyan*) with the PqsD homodimer (*gray*) illustrated as Cα-backbone representations with two views shown. *E,* same as *D*, but only β-strands for PqsB (*red*) and PqsD (*gray*) are shown as schematics.

**FIGURE 5.**
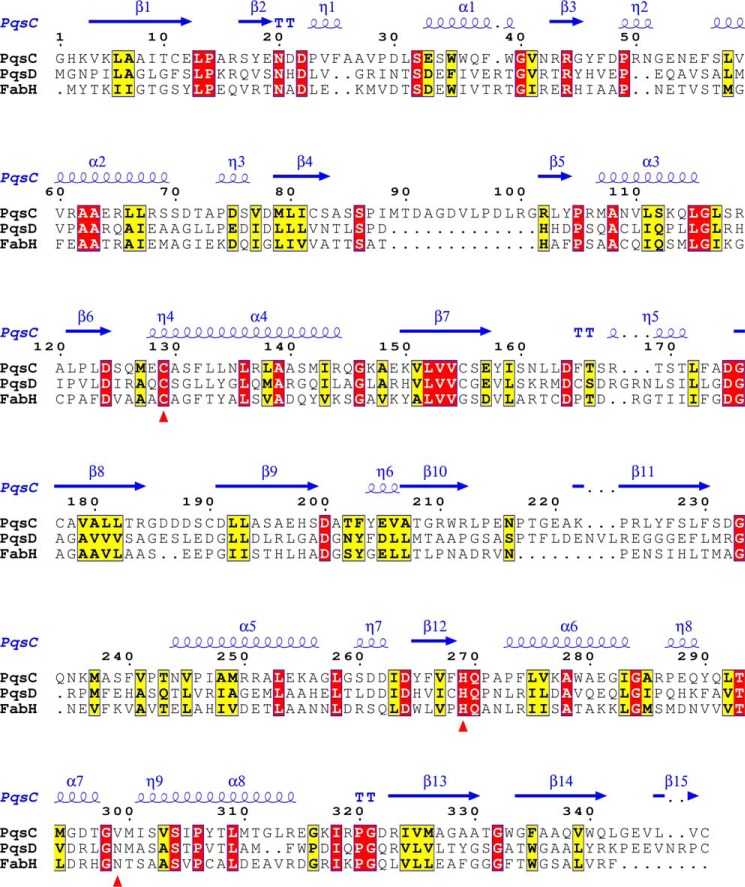
**Alignment of the PqsC amino acid sequence with related enzymes showing the assignments of the PqsC^C129A^ structure at the top (*blue*).** The catalytic residues are marked by *red triangles*. The sequences shown are PqsD (*P. aeruginosa*) and FabH (*E. coli*).

The active-site residues His-269 and the mutated Ala-129 (replacing Cys-129) are clearly defined in the electron density (Fig. 3*A*). In a noticeable difference with other members of the FabH enzyme family, an asparagine amino acid does not form a catalytic triad with His-269 and Cys-129, and in its place a valine (Val-299) is present (Fig. 3*B* shows the superposition of PqsC with the PqsD catalytic triad). Fig. 4*A* shows PqsC^C129A^ superposed onto the PqsD structure (PDB code 3h78) viewed in the area of the active site cleft illustrating significant structural differences. The area of the structure known as the flap in the FabH enzymes (45) is conserved as a short α-helix in PqsD, whereas in PqsC it is formed by a short antiparallel β9-β10 sheet. Directly C-terminal to this, further differences are apparent as the PqsC helix α5 is shorter than the equivalent α-helix in PqsD/FabH. Pro-242 breaks the helix α5 in PqsC and directs an alternate path for the polypeptide compared with FabH/PqsD forming an irregular loop structure, termed loop A, which effectively opens up the active site cleft. Another difference between PqsC and FabH/PqsD structures is an insertion of 14 amino acids in PqsC between strand β4 and helix α3 (Fig. 5; PqsC residues 88–102). This insertion is termed loop B and forms a network of interactions with the PqsC flap and contributes to the dimer interface with PqsB.

Fig. 4*B* illustrates the structure of the PqsBC^C129A^ heterodimer compared with the PqsD homodimer below viewed with the flap region lying close to the PqsD dimer axis (Fig. 4*C*). Fig. 4*D* shows two orientations of a C-α trace of PqsBC polypeptide backbone superposed on the PqsD homodimer. The superposition is driven largely by the similarity between PqsC and PqsD and shows the close relative positioning of the PqsBC subunits compared with the PqsD homodimer. The PqsB β-sheets are displaced by 2–3 Å when overlaid with PqsD (Fig. 4*E*). The PqsBC active site cavity is elongated in the direction of the heterodimer interface where it narrows and extends into PqsB. The gap in the interface is not observed in FabH/PqsD and in part accounts for the smaller ∼2,238 Å^2^ surface area buried by formation of the PqsBC heterodimer compared with the larger value of 3,000 Å^2^ for the FabH/PqsD homodimer.

**FIGURE 6.**
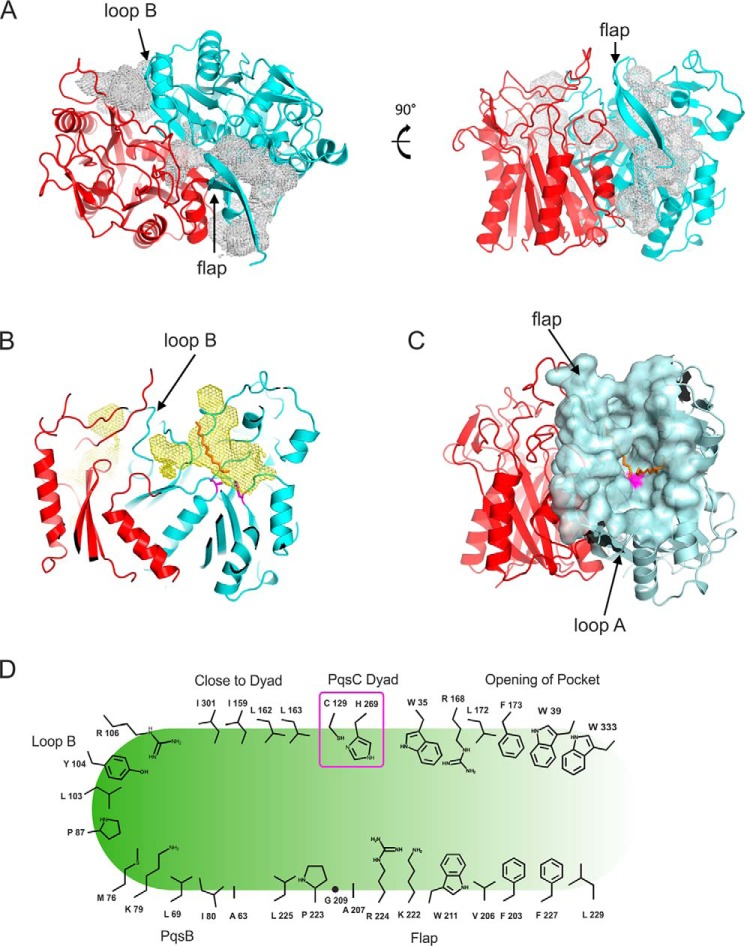
**PqsBC^C129A^ active site pocket.** *A,* two views showing the internal pockets calculated using metapocket shown as *mesh* (*gray*) together with schematic representations of the PqsBC^C129A^ structure. *B,* PqsBC structure is shown with the view clipped such that the PqsC flap is removed to show the relationship between the PqsC catalytic dyad (*purple*) and the PqsC cavity (*yellow mesh*). For perspective, a decyl formate molecule is shown as *sticks* (*orange*) derived from a superposition of the Mtb FabH-decyl formate complex structure (PDB code 2QNZ). *C,* transparent surface representation of the PqsC active site pocket is show (*light cyan*) with residue Ala-129 in *purple*. For perspective, the decyl formate molecules showing the orientation of the proposed entry and exit portals from the FabH-decyl formate complex structure are shown (*orange*). *D,* graphical representation of the PqsBC active site cleft. Residues conserved with CoA-binding sites of FabH/PqsD enzymes are Trp-35 and Arg-168.

The PqsC^C129A^ active site volume is twice as large as those for FabH/PqsD with a calculated volume of 761 Å^3^ compared with 389 Å^3^ for PqsD (PDB code 3h76) and 367 Å^3^ for FabH (PDB code 2QNZ) (calculated using 3vee (46)). Fig. 6*A* highlights internal cavities in the PqsBC structure calculated using metapocket (47) and rendered with PyMOL. The PqsC pocket surrounding the catalytic dyad extends toward the interface with PqsB and is bifurcated with one branch formed by loop B and residues from PqsB and a second branch remains within PqsC in the area of the flap (Fig. 6*B*). Fig. 6, *B* and *C,* shows the decyl-formate ligand from the FabH-decyl formate complex crystal structure (PDB code 2QNZ (45)), which has been superposed onto the PqsBC^C129A^ structure illustrating that the active site pocket extends in two directions from Ala-129 as in FabH (supplemental Movie 1). Fig. 6*D* schematically shows the residues lining the area of the pocket. The binding site of coenzyme A has been characterized in both FabH (PDB code 1hnj) and PqsD (PDB code 3h77) complex crystal structures (27, 48, 49). Two PqsC residues, Trp-35 and Arg-168, homologous to the PqsD (Trp-32 and Arg-151) coenzyme A-binding site are conserved (27, 50). In PqsD these form interactions on either side of the coenzyme A nucleobase suggesting a similar interaction may occur in PqsC (Fig. 6*D*). However, no other similar residues are conserved due to the large changes associated with loss of the N-terminal region of helix α5 in PqsC (Fig. 4*A*).

##### Steady-state Kinetics Suggest Competitive Inhibition of PqsBC by 2-AA

**FIGURE 7.**
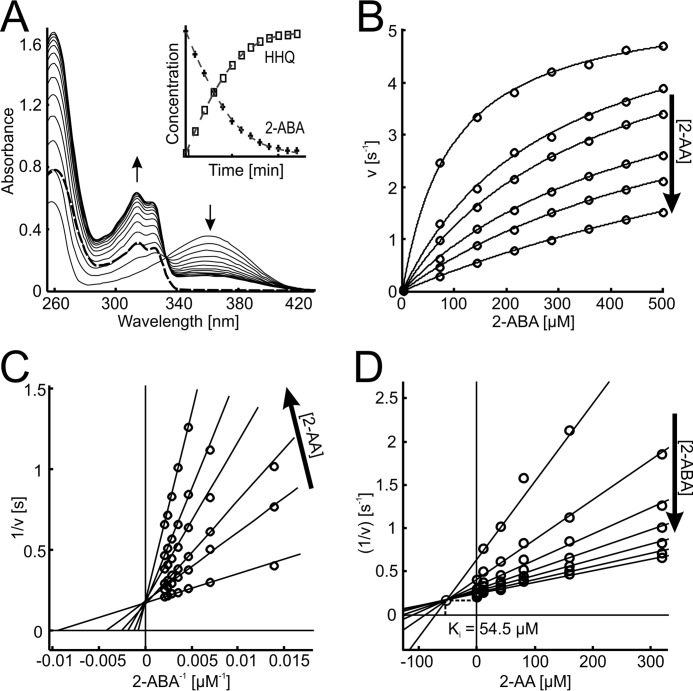
**Steady-state kinetics and competitive inhibition of PqsBC by 2-AA.** *A,* conversion of 2-ABA (λ_max_ at 360 nm) and octanoyl-CoA (absorbing at 260 nm) to HHQ (λ_max_ at 313 nm). 20 nm PqsBC was mixed with 50 μm of each substrate. Spectra were measured every 60 s with a bandwidth of 1 nm. HHQ formation could be observed at 313 nm with a differential extinction coefficient of 6,520 m^−1^ cm^−1^. The remaining absorption above 340 nm after full conversion of the substrates (*bold trace*) is due to scattering and can be eliminated by diluting to 50% in 2-propanol/HCl (*dashed trace*). *Inset,* conversion of 10 μm substrates, monitored at 313 nm (HHQ) and 360 nm (2-ABA) with the determined extinction coefficients. Data interval is 1 min. *B–D,* inhibition of PqsBC activity by 2-AA. *B,* nonlinear fits of measured initial rates. The Lineweaver-Burk plot (*C*) shows the characteristic pattern of competitive inhibition. Using the Dixon plot (*D*), the inhibitor constant *K_i_* of the competitive reaction could be determined graphically. Experiments were performed with 50 μm octanoyl-CoA and 20 nm PqsBC in HEPES buffer, pH 8.2, at 25 °C; *arrows* indicate ascending concentrations.

Using a continuous spectrophotometric assay, initial reaction rates were determined for the PqsBC reaction at substrate concentrations of up to 1 mm 2-ABA and 200 μm octanoyl-CoA. Because a scattering band is emerging at higher concentrations of HHQ (Fig. 7*A*), initial rates were evaluated before the solubility limit of HHQ was reached. Under the conditions of the assay, a *k*_cat_ of 6.8 ± 0.5 s^−1^ was observed, and apparent *K_m_* values of PqsBC were 6.0 ± 0.5 μm for octanoyl-CoA and 105 ± 12 μm for 2-ABA. The His-tagged and untagged protein showed no difference in their kinetic parameters.

To determine whether the various products of the AQ biosynthetic pathway have an effect on PqsBC activity, enzyme assays were performed in the presence of HHQ, PQS, 2-AA, or DHQ. HHQ and PQS as well as DHQ at concentrations up to 50 μm did not affect PqsBC activity. 200 μm DHQ, a concentration that is not reached in *P. aeruginosa* cultures (51), caused an ∼10% drop in activity. In contrast, 2-AA is an efficient inhibitor of PqsBC with an apparent EC_50_ of 46 μm in the *in vitro* assay. It increased the apparent *K_m_* value of the enzyme for 2-ABA, whereas the *k*_cat_ value remained unaffected (Fig. 7, *B–D*), indicating a competitive binding mode with an estimated inhibition constant *K_i_* of 54.5 μm (graphic determination from Dixon plot, Fig. 7*D*) or 56 ± 3.1 μm (global fit according to Equation 4, denoted error is the S.E. of cross-validation).

##### 2-AA Inhibits Biosynthesis of HHQ in Vivo

**FIGURE 8.**
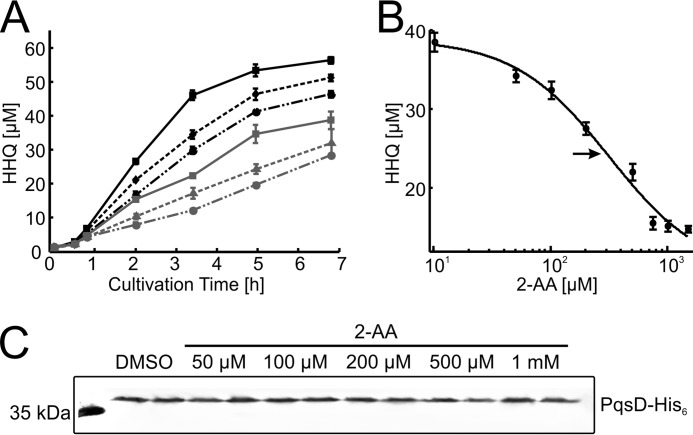
***In vivo* inhibition of HHQ biosynthesis by 2-AA.** Cultures of *P. putida* KT2440 [pBBR::*pqsABCD*-his] were grown in the presence of 1 mm anthranilic acid (as precursor for HHQ synthesis) and various concentrations of 2-AA. 2-AA does not affect growth of *P. putida* KT2440, and it is not utilized as a carbon source. *A*, HHQ contents of culture samples, as determined by HPLC. *Black solid line,* DMSO control; *black dashed line,* 50 μm; *black dash-dotted line,* 100 μm; *gray solid line,* 200 μm; *gray dashed line,* 500 μm; *gray dash-dotted line,* 1 mm 2-AA. *Errors* reflect standard errors (S.E.) of three biological replicates. *B,* dose-response plot, using cultures sampled 3.5 h after inoculation. The *arrow* indicates the calculated EC_50_ of 319 ± 38 μm. *C,* immunodetection of PqsD-His_6_ in Western blots of cell extract supernatants confirms synthesis of the Pqs proteins. Cell extracts were from cultures (independent duplicates) harvested after 7 h of incubation. Each lane contained 50 μg of total protein.

In view of the observed *in vitro* inhibition of PqsBC activity by 2-AA, which is produced by planktonic *P. aeruginosa* cultures in concentrations of up to 200 μm (52), we used a recombinant strain of *P. putida* constitutively expressing the *pqsABCD* genes for testing whether exogenously added 2-AA can inhibit AQ biosynthesis *in vivo*. Any measured inhibitory effect on HHQ biosynthesis in this strain would be caused by inhibition of the biosynthetic proteins. Immunodetection of PqsD, the last protein translated from the polycistronic mRNA, in total cell proteins demonstrated that exposure to 2-AA did not alter the production of Pqs proteins by the cells. 2-AA inhibited HHQ biosynthesis by *P. putida* KT2440 [pBBR-*pqsABCD*], with an EC_50_ value of 319 ± 38 μm (Fig. 8).

Inhibition of HHQ synthesis by 2-AA was observed before for *P. aeruginosa* and was in part attributed to down-regulation of *pqsABCDE* expression by inhibition of the transcriptional regulator PqsR (MvfR). However, post-transcriptional interference of 2-AA with HHQ biosynthesis was also proposed (22). Our *in vivo* and *in vitro* data suggest that competitive inhibition of PqsBC accounts for the post-transcriptional inhibition. Because the IC_50_ of 2-AA of ∼370 μm in *P. aeruginosa* PA14 (indicated by the relative activity of the *pqsA* promoter (22)) is in a similar range as the EC_50_ value determined for the recombinant *P. putida* strain used in this study, the effect of 2-AA on PqsBC likely dominates over its effects on *pqsABCDE* transcription.

##### Octanoylation of PqsBC Has a Major Effect on Binding of the Second Substrate

The apparent Michaelis constant of PqsBC for 2-ABA is rather high, at least when compared with the *K_m_* values of PqsA, PqsD, and PqsH (18, 19, 26). This led us to analyze the binding equilibria of PqsBC with 2-ABA, as well as with its competitive inhibitor 2-AA. Usefully, it turns out that the fluorescence characteristics of 2-AA and 2-ABA, both containing a 2-aminobenzoyl moiety, are strongly influenced by their molecular environment such that the increase in fluorescence upon protein binding could be used for determination of the binding affinities to PqsBC.

The *K_D_* value of about 370 μm determined for the complex of PqsBC with 2-ABA (Table 2) suggests that the affinity of non-octanoylated PqsBC for 2-ABA is even lower, more than 3-fold, than expected from the corresponding apparent *K_m_* value. Exchange of Cys-129 by Ala or Ser had only a minor effect on the *K_D_* value, indicating that this position does not play a crucial role in the binding of 2-ABA. *K_D_* values of the PqsBC·2-AA complex were roughly 2-fold lower, which matches the observed ratio of *K_m_* (2-ABA)/*K_i_*(2-AA) of 2:1.

**TABLE 2:** **Dissociation constants of PqsBC in complex with 2-ABA or 2-AA** *K_D_* values were deduced from fluorimetric titrations (1–10 μm PqsBC and 0–3 mm 2-AA or 2-ABA). Errors reflect standard errors of mean (S.E., *n* ≥3). Titrations were carried out in the absence (−) or presence (+Oct-CoA) of a 10-fold excess of octanoyl-CoA over PqsBC. The values of the H269A and C129S proteins are possibly affected by minor residual activity (*k*_cat_ (H269A) = 0.0005 s^−1^ and *k*_cat_ (C129S), below detection limit of the spectrophotometric assay).

Protein	*K_D_* (2-ABA)		*K_D_* (2-AA)	
	−	+Oct-CoA	−	+Oct-CoA
	μ*m*		μ*m*	
PqsBC	368 ± 19	–*^a^*	161 ± 8	5 ± 1
PqsBC^H269A^	198 ± 11	166 ± 12	117 ± 6	88 ± 5
PqsBC^C129A^	330 ± 30	312 ± 38	158 ± 12	141 ± 19
PqsBC^C129S^	411 ± 43	420 ± 45	170 ± 10	177 ± 8
PqsBC^V299N^	NB*^b^*	–*^a^*	NB*^b^*	89 ± 4

*^a^* Experiment was not possible due to turnover.

*^b^* No binding or *K_D_* values above detection limit (estimated to >1 mm).

**FIGURE 9.**
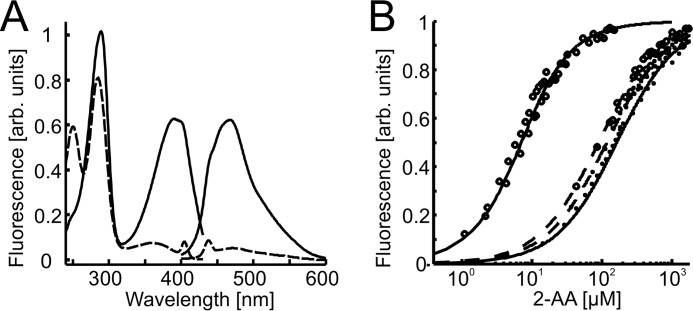
**Equilibrium binding of 2-AA to PqsBC proteins in apo- and octanoylated forms.** *A,* fluorescence excitation and emission spectra of 2-AA (1 μm) in unbound (*dashed line*) and protein-bound (20 μm PqsBC, *continuous line*) form. *B,* titration data and curve fittings of 5 μm PqsBC (*continuous lines*) or PqsBC^H269A^ variant (*dashed lines*) with 2-AA in the presence (*circles*) or absence (*dots*) of 10 μm octanoyl-CoA. Data suggest an increase of binding affinity to 2-AA of the acyl-protein in the wild-type but not in the H269A variant (numerical data provided in Table 2).

The exceedingly high *K_D_* value of the PqsBC·2-ABA complex and the discrepancy between equilibrium binding and kinetic data led us to hypothesize that octanoylation at Cys-129 of PqsC (20), which, based on the current understanding of FabH-like reactions is proposed to be the initial step of the reaction, modulates the affinity of the enzyme for 2-ABA. Assuming that 2-AA binds to PqsBC and to octanoyl-PqsBC in the same way as 2-ABA, we used 2-AA for probing the effect of PqsBC acylation on the affinity for the second substrate. As illustrated in Fig. 9*B*, octanoyl-PqsBC has an ∼35-fold higher affinity for 2-AA than the unloaded enzyme. However, such a change in affinity was not observed for the PqsBC^C129S^ and PqsBC^C129A^ proteins incubated with octanoyl-CoA, supporting the notion that covalent binding of the octanoyl moiety to Cys-129 increases the binding affinity of PqsBC for 2-AA and, most probably, also for 2-ABA. Octanoylation of PqsBC^C129S^ was not observed.

##### His-269 of PqsC Modulates 2ABA/2-AA Binding

Interestingly, replacement of His-269 in PqsC by alanine resulted in significantly altered binding properties of the PqsBC^H269A^ protein for both 2-ABA and 2-AA. The affinity of the (non-acylated) mutant protein was 1.5–2-fold higher than that of wild-type PqsBC for both compounds, although octanoylated PqsBC^H269A^ did not show the increase in affinity for 2-AA that was observed for the octanoylated wild-type enzyme (Fig. 9*B* and Table 2). Again, assuming that the interaction with 2-AA reflects 2-ABA binding, these findings suggest that His-269 acts as modulator, which, depending on the absence or presence of an octanoyl moiety in the active site, has restricting or promoting effects, respectively, on binding of the second substrate. However, when comparing different structures of cysteine-loaded and unloaded FabH-like enzymes, no distinct structural change of the catalytic histidine is observable, suggesting that electronic rather than spatial changes drive the observed affinity shift.

##### Octanoylation of Cys-129 of PqsC Is Independent of His-269

**FIGURE 10.**
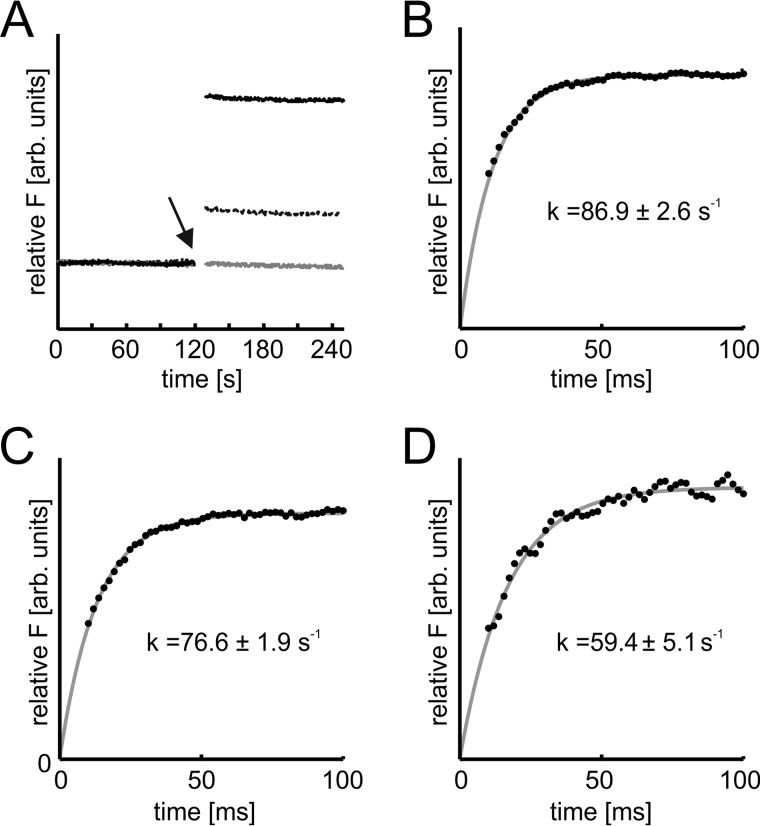
**Role of His-269 in octanoylation of PqsBC, monitored by transient kinetics.** *A,* time-dependent measurement of 2-AA fluorescence (λ_Ex_ = 400 nm, λ_Em_ = 460 nm, 0.5-s time resolution) in complexes with PqsBC proteins. Octanoylation (addition of 10-fold molar excess of octanoyl-CoA indicated by *arrow*) shifts the binding equilibrium of 2-AA and PqsBC (*solid black line*). The effect was reduced in PqsBC^H269A^ (*dashed black line*), and addition of octanoyl-CoA to PqsBC^C129A^ did not change 2-AA fluorescence (*gray line*). Enzyme and 2-AA concentrations were varied between 1–20 and 1–100 μm respectively. *B–D,* octanoylation of PqsBC proteins, analyzed with stopped-flow fluorimetry. *B,* PqsBC octanoylation probed with 2-AA. *C,* PqsBC^H269A^, probed with 2-AA. *D,* PqsBC^H269A^, probed with 2-ABA. Assays were conducted at 6 °C, detector response time was 10 ms; enzyme concentration was varied to achieve sufficient signal to noise ratio. Rate constants were calculated assuming a first order reaction characteristic. *Denoted errors* are standard errors of cross-validation.

In previous studies of FabH and FabH-like proteins, a possible involvement of the active-site histidine in deprotonation of the cysteine to act as a nucleophile in the initial acylation step has been discussed (28, 49). In the case of PqsBC, the observed competitive inhibition of the reaction by 2-AA and the high affinity of 2-AA for the octanoylated protein suggested that this compound could be used as a fluorescence probe to monitor the acylation step in a time-resolved setup (Fig. 10*A*). The binding velocity of 2-AA to PqsBC was faster than resolvable by the stopped-flow instrument (∼5 ms detection limit), which was a prerequisite for using 2-AA as a probe for determination of the octanoylation rate. From single turnover stopped-flow fluorimetric measurements (Fig. 10, *B–D*), rate constants of *k*_WT_ = 86.9 ± 2.6 s^−1^, *k*_H269A_ = 76.6 ± 1.9 s^−1^, and *k*_H269A_(2-ABA) = 59.4 ± 5.1 s^−1^ were deduced for the octanoylation reaction in wild-type PqsBC, the PqsBC^H269A^ variant, and PqsBC^H269A^ when probed with 2-ABA instead of 2-AA, respectively. If the His-269 residue were important for activation of the catalytic Cys-129, more drastic differences in the acylation rates would have been expected.

##### Amino Group of 2-ABA Functionally Replaces the Canonical Catalytic Asparagine

The catalytic cysteine accepts the acyl moiety, while the histidine and asparagine residues in the active site of FabH-like enzymes have been shown to interactively mediate the decarboxylation of the second reactant (malonyl-ACP in FabH and PqsD) (19, 48). In *E. coli* FabH, both residues are required for decarboxylation activity (48). Interestingly, exchange of His-269 to alanine in PqsBC nearly abolished catalytic activity (*k*_cat_ = 0.0005 s^−1^, corresponding to a more than 10,000-fold decrease in activity). This is in striking contrast to FabH, where replacement of the catalytic histidine resulted in only a 10-fold decrease in activity (48). Because the PqsBC^H269A^ protein still undergoes the acylation step (see above), the histidine clearly is essential for a later step of the PqsBC-catalyzed reaction, which apparently does not depend on participation of an Asn residue.

2-ABA, the substrate of PqsBC, in contrast to malonyl-CoA/-ACP, features an amino group in close proximity to the β-keto carbonyl, which, as suggested by the increased stability of 2-ABA over its desamino analog BA in aqueous solutions, influences the chemical properties of the β-keto acid moiety. We hypothesized that in the PqsBC catalytic cycle, the amino group of 2-ABA might functionally replace the amide group of the missing active-site asparagine. To test this hypothesis, we analyzed the conversion of BA by PqsBC. Using octanoyl-CoA consumption as a readout for the reaction, a greatly diminished reaction velocity of 0.02 s^−1^ was observed.

As a complementary approach, we reintroduced the asparagine into the active site of PqsBC. The crystal structure reveals Val-299 occupies an identical position to the corresponding asparagine residue in PqsD (see Fig. 3*B*), and no steric hindrance could be predicted for the amide group extension. PqsBC^V299N^ has a very low activity toward 2-ABA (*k*_cat_ = 0.013 s^−1^). Because the observed *K_D_* value of the complex with 2-AA was 20-fold higher compared with the wild-type enzyme (Table 2), binding of the 2-aminophenyl moiety possibly is spatially impaired, leading to a substrate orientation unfavorable for catalysis. However, BA was converted with a 14-fold increased *k*_cat_ of 0.18 s^−1^ by PqsBC^V299N^. Taken together, the data strongly support a model in which 2-ABA itself supplies the asparagine-equivalent amino function that assists in catalysis of the decarboxylation step.

##### Substitution of Val-299 by Asparagine Impacts Substrate Specificity

Besides condensing anthraniloyl-CoA and malonyl-CoA or malonyl-ACP, PqsD can also mediate the condensation of anthraniloyl-CoA and 3-ketodecanoic acid to form HHQ (53). If PqsBC catalyzed the decarboxylative condensation of octanoyl-CoA and malonyl-CoA, 3-ketodecanoyl-CoA would be formed, so PqsBC might provide a substrate for PqsD-mediated HHQ synthesis. We therefore analyzed PqsBC and PqsBC^V299N^ with respect to their activity toward malonyl-CoA. Although the turnover number of wild-type PqsBC with malonyl-CoA was only 0.0005 s^−1^ and the PqsBC^H269A^ protein was even less active, the V299N variant showed a 5-fold higher activity. Thus, substitution of the active-site Asn by Val, among other adaptations, not only sets PqsBC apart from the canonical FabH-like enzymes, but also reduces the cross-reactivity with malonyl-CoA. With respect to AQ production by *P. aeruginosa*, however, the activity of both PqsBC and PqsBC^V299N^ with malonyl-CoA is far too low to be considered an efficient alternative path for providing a 3-ketodecanoyl moiety for PqsD-supported synthesis of HHQ.

## Discussion

PqsBC, a key enzyme in the biosynthesis of the quorum-sensing signal molecules HHQ and PQS and a member of the β-ketoacyl-ACP-synthase III family of condensing enzymes, has unique features of a heterodimeric subunit composition and the lack of the canonical active-site asparagine. The data presented provide the first crystal structure of PqsBC, and by combining structural data with kinetic and spectroscopic experiments, we were able to characterize individual reaction steps.

Although catalytic involvement of the PqsC subunit has been demonstrated in a previous study by identifying the acylation of Cys-129 (20), the role of PqsB was enigmatic. The crystal structure of PqsBC^C129A^ shows that PqsB has no catalytic residues, and large differences occur in the overall structure compared with PqsC and FabH. The orientation of PqsC and PqsB subunits forms a novel dimer with a pseudo-2-fold symmetry that is unexpectedly similar to the homodimer in FabH-like proteins, and it is likely that PqsB has a scaffolding role in formation of the heterodimer.

The structure of PqsC reveals major structural alterations in the area of the entry to the active site. The shortened PqsC helix α5, a prominent difference to PqsD and FabH, widens the entry to the catalytic site and provides an extended cavity that could accommodate a bicyclic molecule such as the reaction product HHQ.

Within the catalytic cavity of PqsC, the distance and the conformation of Cys-129 and His-269, the two remaining canonical catalytic residues of FabH-like enzymes, are conserved, pointing toward conserved functions in the catalytic mechanism. Compared with malonyl-ACP, the second substrate of FabH, the equivalent substrate 2-ABA of PqsBC (Fig. 1) has the advantage of UVA-absorbing and distinct fluorescent properties that allowed a series of experiments that provided insight into the reaction mechanism. Moreover, the spectroscopic properties of 2-AA, which we identified as a potent competitive inhibitor of PqsBC, enabled us to separately address the initial acylation step. Octanoylation of PqsBC induced a significant increase in affinity for 2-AA, which, albeit less pronounced, could also be measured for 2-ABA binding by the octanoyl-PqsBC^H269A^ protein. Even though similar effects have not been demonstrated for other FabH-like enzymes, such an affinity shift caused by the loading step could be a common trait of FabH-like enzymes to prevent premature binding of the second substrate. Equilibrium titrations suggested that His-269 is a major factor for the acylation-dependent affinity shift.

With respect to the role of the catalytic histidine in the acylation reaction, contradictory models on its influence on the catalytic cysteine in FabH (48, 49) and chalcone synthase (54, 55), a closely related polyketide synthase, have been discussed. In PqsBC, the velocity of octanoylation was virtually the same for the wild-type protein and the PqsBC^H269A^ variant, indicating that the histidine is not required for activation of the acyl-accepting cysteine. Given the similar conformations and distances between the catalytic histidines and cysteines in PqsC and FabH and the fact that Cys-129 of PqsC, like Cys-112 in FabH, is located at the N terminus of an α-helix (Fig. 5), it is likely that the α-helix dipole effect reduces the p*K_a_* value of Cys to facilitate formation of the thiolate ion necessary for transacylase activity, as suggested by Davies *et al.* (48) for FabH. Also, a proton relay mechanism, as hypothesized by Qiu *et al.* (49), seems unlikely for PqsC.

**FIGURE 11.**
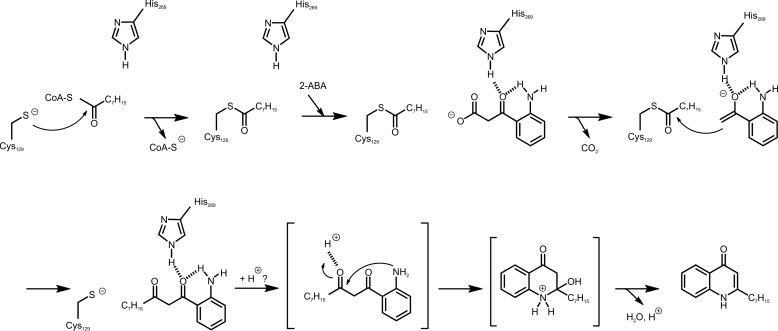
**Proposed catalytic mechanism of PqsBC toward the formation of the 1-(2-aminophenyl)decane-1,3-dione intermediate and its reaction to HHQ.** For details, see text.

The major question of how PqsBC is functional without the catalytic asparagine was addressed with the desamino-substrate analog BA and by reintroducing the asparagine into the protein. Whereas for the wild-type protein the activity toward BA was about 300-fold lower than toward 2-ABA, the PqsBC^V299N^ variant performed significantly better with BA than with 2-ABA. As demonstrated previously for 2-AA (56), the amino group of 2-ABA is hydrogen bonded to its β-keto group (Fig. 11). This intramolecular hydrogen bond replaces the hydrogen bond formed by the active-site Asn of FabH with the thioester carbonyl of malonyl-ACP. Taken together with the observed His-269-dependent binding effect, which (as suggested by equilibrium binding experiments) does not seem to distinguish between 2-ABA and 2-AA, we propose that the amine of the substrate and the histidine's imidazole activate the β-keto oxygen by hydrogen bonding, which promotes decarboxylation and stabilizes the enolate required for nucleophilic attack at the acyl-cysteine (Fig. 11). The reaction product of the subsequent decarboxylative Claisen condensation would be 1-(2-aminophenyl)decane-1,3-dione, which continues to react to form HHQ via intramolecular enamine formation. Even though the active site offers enough space for this consecutive step, the way in which the putative intermediate probably is bound (Fig. 11) renders an on-site ring closing reaction unlikely, because activation of the carbonyl for the subsequent cyclization and elimination step should rather involve a general acid that provides a proton to the keto group that is distal to the aromatic ring. Moreover, the cyclization would require a conformational change within the substrate to bring the amino group in proximity to the distal carbonyl carbon. Given a spatial proximity of the amine and the 3-carbonyl, ring closure could also occur non-enzymatically. Taken together, this work significantly increases our knowledge of the structure, enzyme mechanism, and inhibition of PqsBC, and in a broader dimension, the work has implications for the development of novel therapeutics that control *P. aeruginosa* infection by attenuating virulence gene expression.

## Author Contributions

S. F. and P. W. conceived the study. S. F., J. E., and P. W. coordinated the study. S. L. D., S. F., and J. E. wrote the paper. S. L. D. optimized the synthesis of 2-ABA, prepared PqsBC, and designed, performed, and analyzed all spectroscopic, kinetic, and physiological experiments (Figs. 7–11 and Table 2). F. P. and M. S. performed crystallization, and I. D. and C. L. performed data collection. J. E. performed molecular replacement calculations and density modification. C. L. performed data processing and structure refinement. U. H. prepared 2-aminobenzoylacetate and its precursor, performed all MS and NMR analyses, and contributed to the mechanistic discussion. All authors reviewed the results and approved the final version of the manuscript.

## Supplementary Material

Supplemental Data

## Footnotes

3 The abbreviations used are:

QS: quorum sensing
2-AA: 2-aminoacetophenone
2-ABA: 2-aminobenzoylacetate
2-ABA-CoA: 2-aminobenzoylacetyl-CoA
AQ: 2-alkyl-4(1*H*)-quinolone
BA: benzoylacetate
CV: column volume
DHQ: 2,4-dihydroxyquinoline
HHQ: 2-heptyl-4(1*H*)-quinolone
PQS: *Pseudomonas* quinolone signal
TEV: tobacco etch virus
PDB: Protein Data Bank
ACP: acyl carrier protein.
