# Supplementary material for: PqsBC, a Condensing Enzyme in the Biosynthesis of the Pseudomonas aeruginosa Quinolone Signal: CRYSTAL STRUCTURE, INHIBITION, AND REACTION MECHANISM
Source: J Biol Chem. 2016 Jan 25;291(13):6610–24. doi: 10.1074/jbc.M115.708453 (PMC4807248; doi:10.1074/jbc.M115.708453)
Supplement: Supplemental Data [file supp_291_13_6610__index.html]

PqsBC, a condensing enzyme in the biosynthesis of the Pseudomonas aeruginosa quinolone signal: crystal structure, inhibition, and reaction mechanism — PqsBC, a Condensing Enzyme in the Biosynthesis of the Pseudomonas aeruginosa Quinolone Signal — Structure and Function of the Condensing Enzyme PqsBC — Supplemental Data 

# PqsBC, a Condensing Enzyme in the Biosynthesis of the *Pseudomonas aeruginosa* Quinolone Signal

## Supplemental Data

- Supplementary movie 1 (.mov, 33.2 MB) - Supplementary movie illustrating the active-site pocket.
